# Molecular Docking Approach for Biological Interaction of Green Synthesized Nanoparticles

**DOI:** 10.3390/molecules29112428

**Published:** 2024-05-21

**Authors:** Pallab Kar, Ayodeji O. Oriola, Adebola O. Oyedeji

**Affiliations:** 1 African Medicinal Flora and Fauna Research Niche, Walter Sisulu University, Mthatha 5117, South Africa; pallabkar.bio@gmail.com; 2 Department of Chemical and Physical Sciences, Walter Sisulu University, Mthatha 5117, South Africa

**Keywords:** molecular docking, nanoparticles, green synthesis, pymol, discovery studio

## Abstract

In recent years, significant progress has been made in the subject of nanotechnology, with a range of methods developed to synthesize precise-sized and shaped nanoparticles according to particular requirements. Often, the nanoparticles are created by employing dangerous reducing chemicals to reduce metal ions into uncharged nanoparticles. Green synthesis or biological approaches have been used recently to circumvent this issue because biological techniques are simple, inexpensive, safe, clean, and extremely productive. Nowadays, much research is being conducted on how different kinds of nanoparticles connect to proteins and nucleic acids using molecular docking models. Therefore, this review discusses the most recent advancements in molecular docking capacity to predict the interactions between various nanoparticles (NPs), such as ZnO, CuO, Ag, Au, and Fe_3_O_4,_ and biological macromolecules.

## 1. Introduction

One of the most active areas of materials science research nowadays is nanotechnology. Material scientists have spent the last fifty years researching various applications for nanoparticles (NPs) and nanostructured materials in the biomedical and healthcare industries [1]. The size of the nanoparticles, distribution, and form can all contribute to their new or enhanced properties. The subject of nanotechnology has advanced significantly in recent years, and several methods have been developed to produce precisely sized and shaped nanoparticles according to particular requirements [2]. Nanomaterials and nanoparticles are finding more and more uses every day. The production, characterization, investigation, and use of nanoscale (1–100 nm) materials for scientific advancement is known as nanotechnology. It works with materials where structures show notably novel and improved physicochemical, chemical, and biological properties, phenomena, and use due to their nanoscale size. Compared to macro-sized materials, nanoparticles have a greater surface area due to their smaller size. Size, shape, composition, crystallinity, and morphology are the primary determinants of the intrinsic properties of metal nanoparticles [2]. Due to their small size, nanoparticles differ from the bulk form of the same substance in many ways, opening up new possibilities for biosensors, biomedicine, and bionanotechnology [3]. In addition, medicine is using nanotechnology to diagnose conditions, distribute therapeutic drugs, and create cures for a wide range of illnesses. Due to its immense power, nanotechnology has the potential to be used in medicine for early disease detection, treatment, and prevention. It also offers great promise for the design and development of many new product types [4]. Computational analyses have been applied to electronic structure methods using molecular docking [5], density functional theory calculations, Monte Carlo, kinetic mean-field model, and molecular dynamics simulations to obtain additional insights into the interactions and dynamics of nanoparticles within biological systems [6,7,8,9]. Statistical modeling and machine learning techniques, in particular, are two contemporary molecular modeling methodologies that have been applied to the prediction of NP-biomolecule interactions. Molecular docking has garnered increased attention recently because of its computational ability to predict the interaction and binding energies between nanoparticles and macromolecules. So, the objective of this paper is to showcase the latest developments in molecular docking’s ability to anticipate how different NPs, including ZnO, CuO, Ag, Au, and Fe_3_O_4_, would interact with biological macromolecules.

## 2. Methodology

An extensive literature search was conducted for articles published over the last twenty-five years (2000–2024) in scientific databases, such as Elsevier, Google Scholar, Hindawi, Scopus, PubMed, ScienceDirect, Springer Nature, Taylor and Francis, and Wiley Online. The search keywords and phrases included “nanotechnology”, “metal nanoparticles”, “green synthesis approach to nanoparticles preparation”, “molecular docking”, and “software utilized in nanoparticles molecular docking”. The inclusive criteria of the study were “biological approach to nanoparticles synthesis”, “natural products derived metal-based nanoparticles”, “in silico studies on nanoparticles”, and “in silico properties of metallic nanoparticles”. The exclusive criteria included “in vitro and in vivo biological studies on green synthesized metallic nanoparticles”.

## 3. Concept of Nanotechnology

While nanomaterials have been around for a while, the last 20 years have seen substantial progress in the field of nanoscience. Nobel laureate Richard Feynman introduced the concept of nanotechnology in his well-known speech at the California Institute of Technology on 29 December 1959. The concept of nanoparticles was covered by Richard Feynman in his 1960 paper “*There is plenty of room at the bottom*”. He observed that all books ever written could fit inside a cube with sides that were 0.02 inches long if all that was needed to hold a bit of information was only 100 atoms [10]. Norio Taniguchi first defined the term “nanotechnology” in 1970. According to him, material processing, deformation, separation, and consolidation performed by a single atom or molecule are the main uses of nanotechnology. Furthermore, in 1980, another technologist named K. Eric Drexler promoted the technological significance of nanotechnology [11]. The primary distinction between bulk and nanoscale characteristics is the nature of particles in the nano dimension. Nanoparticles are employed in a variety of fields, such as electrical, biological, textile, and chemistry, where the size and shape of colloidal metal particles are important for a variety of applications, such as the creation of biocomposites, electronic devices, magnetic materials, wound healing, and antimicrobial gene expression [3,4]. The size and shape of the particles determine the optical and catalytical electromagnetic properties of noble metal colloids.

## 4. Green/Biological Approaches for the Synthesis of Metal Nanoparticles

**Figure 1:**
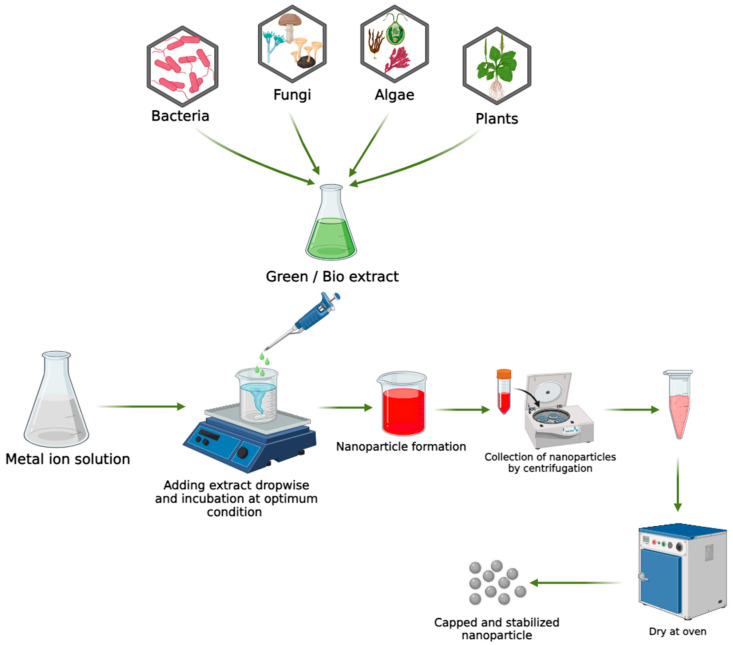
The schematic diagram shows the biosynthesis of NPs.

“Green” or “biological” nanoparticle synthesis is the process of creating different types of metal nanoparticles by using bioactive substances such as plant materials, microorganisms, algae, and so on (Figure 1) [12]. Table 1 shows the overall characteristics of metal-based nanoparticles using green/biological approaches. There are many benefits of using green synthesis to create nanoparticles. By using natural reagents rather than dangerous chemicals, this strategy is both environmentally benign and hopeful since it reduces carbon emissions and promotes sustainable development [13]. It has been demonstrated that using this technique can produce nanoparticles that have a smaller environmental impact and better biocompatibility [14]. It is also more sustainable because it uses natural resources and is less expensive [15]. However, it does have certain restrictions. Longer response times are among them, as are difficulties in regulating the size, shape, and polydispersity of nanoparticles [16]. Green synthesis is a promising technique for the synthesis of nanoparticles with potential applications in a variety of disciplines despite these drawbacks [16].

### 4.1. Biological Synthesis Using Microorganisms

Silver (Ag), gold (Au), copper oxide (CuO), iron oxide (Fe_3_O_4_), and zinc oxide (ZnO) are nanoparticles that are frequently employed as antibacterial agents [54,55]. Numerous investigations have demonstrated the biocidal activity of several nanoparticles against Gram-positive and Gram-negative bacteria, fungi, and viruses [56]. The antimicrobial properties of metallic nanoparticles (NPs) are significantly influenced by their high specific surface area, high surface-to-volume ratio, and nanoscale size. These features enable a strong interaction with membranes of microorganisms, causing disruption and subsequent penetration into cells, damage to internal cellular structures, and, ultimately, cell death [57]. The processes via which metallic nanoparticles can circumvent antibiotic resistance. It has been previously reported that bacteria such as *Enterococcus* sp., *Escherichia coli*, and *Ochrobactrum anthropic*-based metal NPs have potential photocatalytic characteristics, as well as antibacterial and anticancer activity [58,59,60,61].

### 4.2. Biological Synthesis Using Fungi

Fungal-based nanoparticle biogenesis is a commonly employed method due to its ability to produce monodisperse particles with a wide range of sizes, shapes, and chemical compositions. Because they can be easily handled and have multiple enzymes in their cells, fungi are thought to be great options for producing metal and metal sulfide nanoparticles [62]. The mycelia surface is where the nanoparticles were formed. The enzymes present in the mycelia’s cell wall are responsible for fostering the electrostatic interaction between negatively charged carboxylate groups and silver ions, which is what initially traps the Ag+ ions on the fungal surface of cells, according to the data analysis and solution recording. These nuclei then increase when more Ag ions are reduced and accumulated on them. The TEM findings show that certain silver nanoparticles are present on the cytoplasmic membrane as well as inside of it. The findings demonstrated that the quantity of Ag ions that went through the cell wall was decreased by enzymes on the cytoplasmic membrane and within the cytoplasm. Other possibilities include some silver nanoparticles diffusing through the cell wall and then becoming trapped in the cytoplasm [63,64]. The accumulation of the gold nanoparticles was significantly impacted by the incubation temperature. It was discovered that the particle formation rate accelerated at higher temperatures [64]. It has been observed that *Verticillium luteoalbum* produces 20–40 nm-sized gold nanoparticles [65]. Metal nanoparticles based on *Aspergillus terreus* and *Penicillium brevicompactum* have been shown to exhibit antibacterial and cytotoxic properties [66,67].

### 4.3. Biosynthesis of NPs Using Algae

Algae contain large amounts of polymeric polymers, which have the potential to hyper-accumulate heavy metal ions and transform them into malleable forms. Algal extracts frequently contain pigments, sugars, proteins, minerals, polyunsaturated fatty acids, and other bioactive materials, including antioxidants that act as reducing, capping, and stabilizing agents [68]. In comparison to their biosynthetic counterparts, NPs photosynthesize at a faster rate. When producing bionanomaterials like metallic nanoparticles, an environmentally acceptable method uses live or dead algae as model organisms [69]. The most studied noble metals for algal NP synthesis, whether intracellularly or extracellularly, are Ag and Au. Metal nanoparticles (NPs) based on *Chlorella vulgaris* and *Nanochloropsis oculata* have been shown to have antibacterial and catalytic properties, and they are also used in Li-Ion batteries [70,71,72].

### 4.4. Biological Synthesis Using Plant Extracts

A plant extract is the material or active component of the required quality that is removed from plant tissue by treatment for a specific use. Plant extracts are combined with a room-temperature metal salt solution to create nanoparticles. Silver, gold, and numerous other metal nanoparticles have been produced using this technique [73]. Many types of plants are used in the biosynthesis of nanoparticles. It is commonly recognized that the quantity and other characteristics of nanoparticles, as well as their rate of generation, are influenced by the kind of plant extract, its concentration, the concentration of metal salts, pH, temperature, and length of contact time. Silver nanoparticles were made using an extract from *Polyalthia longifolia* leaves; the average particle size was approximately 58 nm [43,74,75]. *Coffea arabica*, *Digitaria radicosa*, *Diospyros paniculata*, *Elephantopus scaber*, *Bergenia ciliate*, *Azadirachta indica*, *Anisomeles indica*, *Acacia auriculiformis*, *Clitoria ternatea*, *Emblica officinalis*, *Euphorbia antiquorum*, *Ficus benghalensis* are the few plants that have been documented to have a green synthesis of metal nanoparticles (Ag NPs). The antifilaria, antibacterial, antioxidant, and cytotoxic properties of these were previously assessed [76,77,78,79,80,81,82].

Many more natural products mediated metallic NPs and their applications are presented in Table 2.

## 5. Software Utilized in NPs Molecular Docking

**Figure 2:**
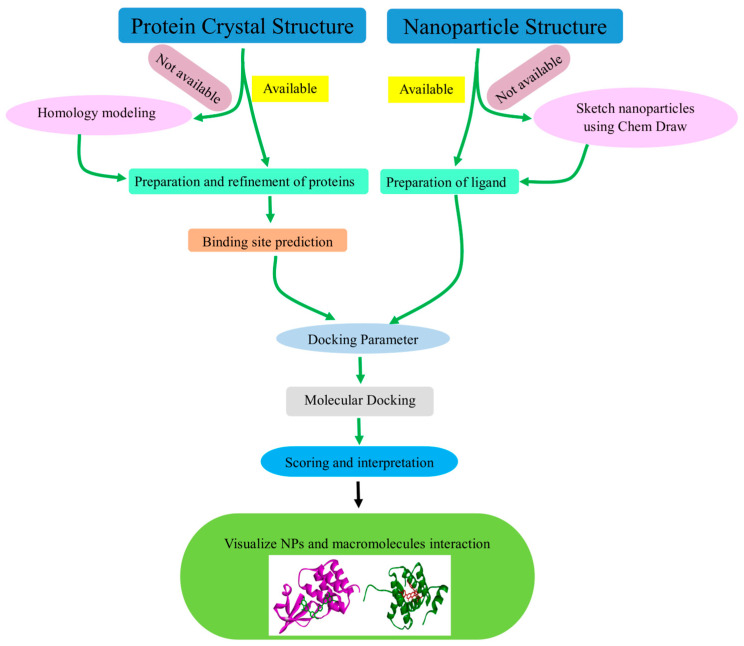
The steps in the simulation of molecular docking for nanoparticles.

In order to obtain an accurate representation of the proposed interaction between the macromolecules and the NPs, the molecular docking simulation process involves multiple stages (Figure 2). The structure of NPs needs to be meticulously constructed to replicate their exact dimensions and shape. The original chemical structure of the nanoparticles (NPs) can be obtained from online resources such as the Cambridge Cluster Database, or it can be manually constructed using a variety of software programs such as Chem Draw version 22.0 [119,120]. It is necessary to optimize the geometry of those NPs by energy minimization, applying the proper methodology and theoretical level. The optimization of NPs has been accomplished in the recent literature by using a multitude of software modules. According to the majority of research, NPs are fewer than 100 nm in size and can be spherical, lattice, or sheet-shaped [121,122,123]. Moreover, a few studies have concentrated on small single units in the angstrom range, which are representative of real-world sizes. Throughout the NPs structure preparation process, it is crucial to accurately mimic the pre-dominant shape and size of the NPs in biological fluids. For example, some NPs, like gold, have a propensity to cluster rapidly in solution [124]. The real structure of the NPs under physiological settings must be carefully constructed, parametrized, and added to the docking program in order to predict the binding mode. Common NPs are already available in a number of databases with experimental data, such as Springer Nature InterNano, Nano-EHS, NanoHUB, and NANO [125]. Typically, one can obtain the relevant protein crystal structure by downloading it from the RSCB Protein Data Bank (PDB). The appropriate software may include SwissModel (https://swissmodel.expasy.org/), Modeler version 3.9 (BIOVIA), and Prime version 4.0 (Schrodinger), which must be used to generate a homology model. The preferred software is used to carry out the docking procedure following the ligand and macromolecule’s preparation. It is important to note that no specialized software is available to manage NP docking simulations. Most of the recent investigations have made use of the AutoDock application [126]. Furthermore, NPs docking has lately been performed on a few online docking sites. These include the Fourier transform-based approach used in HEX 6.3 [127] and the shape complementarity-based technique used in Patchdock [128]. Furthermore, we have seen that a tight docking technique with fixed protein residues was used in most published research. The last stage involves visualizing how NPs and macromolecules interact using a variety of tools, including Pymol, Discovery Studio, iGEMDOCK, and UCSF Chimera [129,130].

## 6. Analysis of Biomacromolecule–NP Interactions Using Molecular Docking

The common categories of medicinally significant nanoparticles and the docking studies published over the past 20 years to forecast their interactions with various biological molecules are described in the section that follows.

### 6.1. Zinc Oxide Nanoparticles

Scientists are becoming more interested in zinc oxide nanoparticles (ZnO NPs) due to their inexpensive cost and low toxicity in bioapplications. According to Sabir, Arshad, and Chaudhari [131], these NPs have a variety of uses in the food additive, cosmetics, rubber, antimicrobial agents, and photocatalysts industries. With a binding energy of −2.93 kcal/mol, zinc oxide nanoparticles utilizing *Cymbopogon citratus* extract demonstrated a good binding relationship between ZnO and DNA gyrase subunit b [132]. Alrabayah and colleagues used *Cestrum diurnum* leaf extract to produce zinc oxide nanoparticles (ZnO NPs) using a green chemistry approach. They discovered that the ZnO NPs exhibited a strong affinity against the major protease, HCOV-229E [133]. Zinc oxide nanoparticles (ZnO NPs) utilizing extract from *Dysphania ambrosioides* showed positive molecular docking interactions with certain proteins in both Gram-positive and Gram-negative bacteria, including AcrAB-TolC in *Escherichia coli* and TagF in *Staphylococcus epidermidis* [134]. ZnO NPs can bind with energies of about −5.44 and −1.56 kcal/mol to human insulin and BSA at subdomain IIIA, respectively [135]. Nonetheless, a different investigation revealed that ZnO NPs may also attach to the IB and IIA subdomains [122]. ZnO NPs have a stronger binding attraction toward AChE and BChE than Fe_3_O_4_ NPs. PbO NPs, however, exhibited the highest affinity for these CNS enzymes, with calculated binding energies of −4.67 and −3.89 kcal/mol, respectively [136]. ZnO NPs can change the expression of C-C motif chemokine 18 (CCL18), NF-κβ, ICAM-1, interleukin 8 (IL8), P-38, cluster of differentiation 35 (CD35), and interleukin 1 beta (IL1B) when they bind to chemokine and specific cellular proteins. P38 (PDB ID: 3W8Q) had the highest binding affinity (−8.81 kcal/mol), while IL-1B (PDB ID: 1I1B) had the lowest (−3.23 kcal/mol). Furthermore, ZnO NPs were studied as α-glucosidase enzyme inhibitors, a popular target for the management of diabetes [137]. When ZnO NPs were generated at the 500 °C calcination temperature, they had the strongest binding energy to this enzyme (−13.64 kcal/mol), with a size of around 32 nm [126].

### 6.2. Copper Oxide Nanoparticles

Copper oxide nanoparticles (CuO NPs), in contrast to other metal oxide nanoparticles, have garnered significant interest for application because of their distinct size-dependent chemical properties, captivating attributes, and inexpensive manufacturing cost [138]. Several intriguing applications of CuO nanoparticles have been demonstrated, including the detection of the H1N1 virus [139], the preparation of antibacterial and antifungal medicines [140], and bioremediation [141]. CuO NPs are in high demand; hence, assessing their nanotoxicity is crucial to ensure their safe use. CuO NPs with binding energies of −12.562 and −8.797 kcal/mol exhibit good interactions with their targets, as demonstrated by the production of CuO NPs from *Acer palmatum* leaf extract. To investigate the antibacterial mechanisms of CuO NP, molecular docking analysis was carried out using DNA Gyrase B from *Staphylococcus aureus* and *Escherichia coli* [142]. Kocabas and colleagues used *Phragmites australis* leaf extract for the synthesis of copper oxide nanoparticles. They carried out in silico molecular docking against the active binding sites of dihydropteroate synthase, thymidylate kinase, and *Staphylococcus aureus* FtsZ, with docking scores of −9.067, −8.048, and −7.349 kcal/mol, respectively [143]. In contrast to the binding of TiO_2_ to HSA into subdomain IIA, the expected binding position of CuO NPs in the same protein was subdomain IIIA, aided by the interaction with Arg472 [144]. The binding mechanism of CuO NPs with the protein targets he1a, sod1, and tp53 was also investigated using molecular docking. However, they showed insignificant binding energies of −1.23, −0.91, and −1.07 kcal/mol, respectively, according to Kumari et al. [145]. These binding energies suggest a weak interaction with the protein because they are near the thermal energy.

### 6.3. Silver Nanoparticles

Ag NPs, or silver nanoparticles, are widely employed as electronic components, ink additives, food preservatives, and antibacterial agents [146,147]. The Bcl-X_L_ protein exhibited positive molecular docking interactions with silver nanoparticles derived from *Elaeagnus pyriformis*, with binding energies of −6.8 kcal/mol and −6.5 kcal/mol, respectively [148]. Banerjee et al. [149] synthesized silver nanoparticles in a different work using fruit extract from *Phyllanthus acidus*. The produced nanoparticles had an inhibitory effect on the inflammatory protein NFκβ, according to an in silico molecular docking analysis, with docking scores of −6.9 and −6.5 Kcal/mol [149]. Among the six different isoforms of cytochrome P450 (CYP), Ag NPs have been demonstrated to bind with great affinity to CYP2C9, CYP2C19, and CYP2D6 in the human body [150]. Ag NPs were examined in tests with a variety of pathogens. Because of its part in the virus’s contagiousness, Ag NPs were docked into the HIV protease enzyme, one of the proteins most targeted for HIV defense [151]. The main amino acids in bacterial proteins that can interact with Ag NPs were also looked into using molecular docking. The quorum sensing systems that are formed by the bacteria *Pseudomonas aeruginosa* are mediated by proteins called LasR, QscR, RhlR, and Vfr-like [152]. The following residues were identified by Ag NP docking into these proteins: RhlR (Tyr72, Trp68), QscR (Arg167, Ala232), LasR (Leu36, Asp73), and Vfr (Lys28) [128]. Additionally, the importance of particular amino acids in the interaction of Ag NPs with RhlI (His52), LasI (Asp73), LasR (Leu159), and RhlR (Trp10) was validated by a different study [153].

### 6.4. Gold Nanoparticles

Gold has a number of uses in medicine, including as gold salts that have antimicrobial properties [154] and as nanoparticles that are used in cancer treatment [155]. Using molecular docking, the relationship between anti-EGFR-iRGD, a tyrosine kinase connected to several cancers, and gold (Au) nanoparticles was also examined. In this interaction, asn845 was the essential amino acid, with a binding energy estimate of −3.5 kcal/mol [156]. According to Al-Radadi [157], gold nanoparticles containing *Commiphora myrrh* exhibited a stronger negative docking score (−3.976 Kcal/mol) when compared to the VEGFR-2 domain. Despite its potential therapeutic significance, we firmly believe that not enough information exists on the mechanism of Au NPs’ interaction with biological macromolecules.

### 6.5. Iron Oxide Nanoparticles

Due to their widespread use in the biomedical industry for labeling, drug transport, magnetic resonance imaging, cellular targeting, and magnetic hyperthermia, iron oxide nanoparticles have attracted much attention. In addition to having low toxicity and decreased sensitivity to oxidation, they offer distinctive magnetic properties and intriguing biocompatibility qualities [158,159]. Yasmin Abo-zeid and colleagues used docking studies to examine the interaction of iron oxide nanoparticles (IONPs) (Fe_2_O_3_ and Fe_3_O_4_) with the spike protein receptor binding domain (S1-RBD) of SARS-CoV-2. The glycoproteins E1 and E2 of the hepatitis C virus (HCV) were also subjected to a comparable docking investigation. These investigations demonstrated the effective interactions between Fe_2_O_3_ and Fe_3_O_4_ and the SARS-CoV-2 S1-RBD, as well as the HCV glycoproteins E1 and E2 [160]. Using molecular docking modeling, the binding energy of lysozyme (PDB ID: 6LYS) with Fe NPs was calculated and determined to be roughly 230.92 kJ/mol [161].

## 7. Conclusions

Nanomaterials have better and more customizable physical, chemical, and biological properties than bulk materials, making them intriguing materials. Based on their size, shape, content, and place of origin, nanomaterials can be categorized. In this review, we discussed the biological and green methods for synthesizing metal nanoparticles and the use of computational methods to evaluate and comprehend the mechanism of nanoparticle-important biomolecule interactions. Molecular docking studies conducted recently have revealed the general pathways by which NPs interact with biological systems. We also investigated the binding energies and the necessary amino acids for binding with particular biological targets. The use of molecular docking techniques to examine the biological activity of nanoparticles is still relatively new. Green manufactured nanoparticles used as biological agents in agriculture and food sectors, as well as the early management of many human diseases, can be facilitated by the application of this molecular docking technique.

## Figures and Tables

**Table 1:** The general properties of biologically or environmentally based metal-based nanoparticles.

Nanoparticle	Natural Source	Size	Shape	Specific Surface Area	Solubility	Optical Properties
ZnO NPs	*Sargassum muticum* (Algae) *Vigna mungo* (Plant) *Prunus bracteopadus* (Plant)	30–57 nm 65 nm 55 nm	hexagonal [17] Spherical [18] Spherical [19]	88.89 m^2^/g [20]	0.3–3.6 mg/L in aqueous medium [21]	Poorly conductive [22]
Cu NPs	*Pseudomonas stutzeri* (Bacteria) *Bifurcaria bifurcate* (Algae) *Gloriosa superba* (Plant) *Coffea Arabica* (Plant) *Thymus vulgaris* (Plant) *Glycine max* (Plant)	50–150 nm 5–45 nm 5–10 nm 20–60 nm 23–94 nm 26.6 nm	Cubical [23] Spherical, Elongated [24] Monoclinic, Spherical [25] Monoclinic [26] Spherical [27] Spherical, Hexagonal [28]	5–10 m^2^/g [29]	At pH 9–11, there is minimal Cu solubility; nevertheless, above pH 11, CuO complexes with hydroxide ions [30]	Highly conductive [22]
Ag NPs	*Bacillus cereus* (Bacteria) *Pseudomonas deceptionensis* (Bacteria) *Aspergillus tamarii* (Fungi) *Fusariumo xysporum* (Fungi) *Pencillium ochrochloron* (Fungi) *Calliandra haemacephala* (Plant) *Musa paradisiaca* (Plant) *Alysicarpus monilifer* (Plant)	4–5 nm 10–30 nm 3–5 nm 5–15 nm 7.7 nm 70 nm 23.7 nm 5–45 nm	Spherical [31] Spherical [32] Spherical [33] Spherical [34] Spherical [33] Spherical [35] Spherical [36] Spherical, Hexagonal [37]	23.81 m^2^/g [20]	Superior solubility in water and extended colloidal stability [38]	Highly reflective [22]
Au NPs	*Chlorella vulgaris* (Algae) *Lemanea fluviatilis* (Algae) *Pogostemon benghalensis* (Plant) *Mentha* (Plant) *Lantana camara* (Plant) *Cannbis sativa* (Plant) *Mimusops elengi* (Plant)	2–10 nm 5–15 nm 10–50 nm 10–100 nm 4–12 nm 10–35 nm 9–14 nm	Spherical [39] Cubic [40] Spherical, Triangular [41] Spherical, Triangular [42] Spherical [43] Spherical [44] Spherical [45]	5.8–107 m^2^/g [46]	AuNPs have great solubility in organic solvents such as toluene [47]	Highly reflective [22]
Fe NPs	*Sargassum muticum* (Algae) *Eichhornia crassipes* (Plant) *Urtica dioica* (Plant) *Mentha spicata* (Plant) *Murraya koenigii* (Plant)	18 nm 20–80 nm 21–71 nm 20–45 nm 59 nm	Cubic [48] Spherical [49] Spherical [50] Crystalline [51] Spherical [52]	14.42 m^2^/g [53]	Insoluble in water and inorganic solutions [53]	Poorly conductive [22]

**Table 2:** Some natural products mediated metallic nanoparticles and their applications.

Natural Source	Metallic Nanoparticles	Application	Reference
Algal extract based			
*Spirulina platensis* *Lyngbya majuscule* *Rhizoclonium hieroglyphicum*	Au NPs	Bio-recovery of accumulated gold (industrial application)	[83]
*Ulva fasciata*	Ag NPs	Biopesticidal application	[84]
*Turbinaria conoides*	Ag NPs	Synthesis of valuable gold nanoparticles for biomedical application	[85]
*Laminaria japonica*	Ag NPs	Bio-recovery of accumulated gold (industrial application)	[86]
*Gelidiella acerosa*	Ag NPs	Biological and biomedical applications	[87,88]
*Cystophora moniliformis*			
*Desmarestia menziesii*			
*Padina tetrastromatica*	Ag NPs	Synthesis of antimicrobial Ag NPs (medicinal application)	[89]
*Sargassum polycystum*			
*Chlamydomonas reinhardtii*	Cadmium sulfide (CdS) bimetallic NPs	Biosensors, photocatalysis, and light-emitting diodes (LEDs)	[90]
*Enteromorpha flexuosa*	Ag NPs	Antimicrobial therapy in modern medicine	[91]
*Pithophora crispa*	Au NPs	Production of semiconductor nanoparticles, including silicon nanoparticles that are employed as bio-indicators in various industrial waste products	[92]
*Gracilaria edulis*	Ag NPs ZnO NPs	Biological/medicinal application as an antimicrobial agent	[87]
Bacterial extract based			
*Bacillus subtilis*	Au-CN_2_^−^	Biosorption removal and concentration of gold from solutions containing residual cyanide (industrial application). Antimicrobial agent	[93,94]
*Bacillus megaterium*	Ag NPs	Biological application as an antibacterial agent against drug-resistant clinical pathogens	[95]
*Bhargavaea indica*	Ag NPs Au NPs	Biotechnology application	[96]
*Escherichia coli*	Ag NPs	Biological application as an antimicrobial agent	[94]
*Lactobacillus plantarum*	MgO NPs	Biomedical and nanotechnology application—cytotoxicity against human leukemia cells	[97]
*L. sporogenes*			
*Nocadiopsis valliformis*	Ag NPs	Biological application as an antibacterial and cytotoxic agent	[98]
*Streptococcus thermophilus*		Biological application as an antibacterial and antifungal agent	[94]
Fungal extract based			
*Agaricus biosporus*	MgO NPs	Useful to stimulate seed germination and the growth of peanut plants	[99]
*Basidiomycetes* sp.	Ag NPs	Biological application as an antibacterial agent	[100]
*Colletotrichum* sp.		Biological application—bactericidal activity against selected human pathogens	[101]
*Neurospora crassa*	Alloy-type Au/Ag bimetallic NPs	NPs stabilization and facile and economical biomass handling	[102]
*Rhizopus oryza*	Gold-nano-bioconjugate	Production of important enzymes, including amylase, lipase, pectinolytic enzymes, and in biodiesel production	[103]
*Trichoderma harzianum*	Ag NPs	Biological application as an antimicrobial	[104]
*Penicillium chrysogenum*	Au-CN_2_^−^	Biosorption—removal and concentration of gold from solutions containing residual cyanide (industrial application)	[93]
*Sargassum fluitans*			
*Pochonia chlamydosporium*	MgCl_2_ NPs	Potential nano-nutrients for plants	[105]
*Aspergillus fumigatus*	MgSO_4_ NPs		
*Aspergillus wentii*	Fe_2_O_3_ NPs FeSO_4_ NPs		
*Curvularia lunata*	Fe_2_O_3_ NPs FeSO_4_ NPs		
*Chaetomium globosum*	Fe_2_O_3_ NPs		
Plant extract based			
*Blumea eriantha*	Ag NPs Fe_2_O_3_ NPs	Biological application as an antioxidant, antibacterial, and cytotoxic agent	[106]
*Buxus wallichiana*	NiO NPs	Biological application as an antioxidant and bactericidal agent	[107]
*Camellia sinensis*	Ni NPs	Industrial application—photocatalysis	[108]
*Citrus sinensis*	ZnO NPs	Biomedical application as an antibacterial agent	[109]
*Clitoria ternatea*	Au NPs	As a stabilizing and reducing agent to reduce the consumption of harmful substances	[110]
*Coffea arabica*	Ag NPs	Biological application as an antibacterial agent	[76]
*Dalbergia sissoo*	MgO NPs	Photocatalysis and biological application as an antibacterial agent	[111]
*Hordeum vulgare*	Ni NPs NiO NPs	Photocatalysis and biological application as an antioxidant agent	[107]
*Moringa oleifera*	Ag NPs	Its antimicrobial and optical properties make it potentially useful in water treatment	[112]
*Myristica fragrans*	Ag NPs	Antibacterial, antifungal, and anticancer activities, thus, may be utilized in the agrochemical and pharmaceutical industries, as well as for biomedical applications.	[113]
*Olea europaea*	Ag NPs	Synthesis of Ag NPs for antibacterial application	[114]
*Phyllanthus emblica*	MgO NPs	Photocatalysis—removal of dye from wastewater. Biological application antibacterial agent.	[115]
*Pisonia alba*	MgO NPs	Biological application as an antifungal agent	[116]
*Platanus orientalis*	Fe_2_O_3_ NPs	Biological application as an antifungal agent against *Aspergillus niger* and *Mucor piriformis*	[117]
*Trigonella foenum-graecum*	Ag NPs	Biological application as an antibacterial and antifungal agent	[118]

## Data Availability

Not applicable.
